# Expression and functional characterization of His-tagged prestin in Chinese hamster ovary cells

**DOI:** 10.1371/journal.pone.0314517

**Published:** 2024-12-02

**Authors:** Ryosei Motoo, Hisashi Sugimoto, Yasunori Donjo, Tomokazu Yoshizaki, Michio Murakoshi

**Affiliations:** 1 Department of Otolaryngology-Head and Neck Surgery, Kanazawa University, Kanazawa, Japan; 2 Faculty of Frontier Engineering, Institute of Science and Engineering, Kanazawa University, Kanazawa, Japan; Icahn School of Medicine at Mount Sinai Department of Pharmacological Sciences, UNITED STATES OF AMERICA

## Abstract

The electromotility of outer hair cell is considered to be based on voltage-dependent conformational changes in the motor protein prestin. The structure and function of prestin have been increasingly examined in recent years. To obtain further information on prestin, a method to stably obtain prestin as the material for this research is required. This study attempted to construct a stable expression system for prestin on Chinese hamster ovary (CHO) cells and its function was evaluated. His-tagged prestin expression vectors were transfected into CHO cells and high-expression clones were obtained by drug selection and limiting dilution method. The expression and activity of prestin were examined by Western blotting and immunofluorescent experiments and by patch clamping. Clones with high fluorescent intensity tended to exhibit bell-shaped non-linear capacitance, suggesting the active function of prestin in such clones. The stable expression and activity of prestin in CHO cells were confirmed in 7 clones out of twelve clones developed. Although expression levels were 1/60-1/30 of those in OHCs, cell line maintaining the stable expression of functional prestin with His tag will be advantageous for future analyses of its structure and function.

## Introduction

Outer hair cells (OHCs) in the mammalian organ of Corti elongate and contract in response to changes in their membrane potential. This OHC motility is generally referred to as electromotility [1, 2]. Electromotility amplifies vibrations of the organ of Corti and this mechanism enables the high hearing sensitivity, wide dynamic range and sharp frequency selectivity of hearing in mammals [3]. The underlying molecular mechanisms are considered to be dependent on voltage, independent of adenosine triphosphate (ATP), and involve conformational changes in a motor protein in the lateral plasma membrane of OHCs [4, 5]. In 2000, this motor protein was identified in the gerbil cochlea and termed prestin [6]. Since its identification, prestin has been extensively examined to elucidate the characteristic behavior of OHCs.

Mammalian cells transfected with prestin exhibit electromotility [1, 6], voltage-dependent non-linear capacitance (NLC) [6–8], and force generation [7]. Furthermore, the amino and carboxyl termini of prestin were detected on the intracellular side [8] and intracellular anions act as voltage sensors of prestin [9]. In contrast, OHCs isolated from prestin knockout mice lacked electromotility and showed a 40- to 60-dB loss in cochlear sensitivity *in vivo* [10]. These findings indicate that prestin is the motor protein in OHCs.

Prestin consists of 744 amino acids with a molecular weight of approximately 81.4 kDa [6]. Studies performed in 2021 and 2022 reported the single particle cryo-electron microscopic structure of prestin [11–14]. However, the mechanisms by which prestin functions at the molecular level have yet to be elucidated. To obtain further information on the structure and function of prestin, a method to obtain a large amount of prestin as the material for this research is required. Therefore, the present study attempted to construct stable expression systems for histidine (His)-tagged prestin on Chinese hamster ovary (CHO) cells. The expression and activity of prestin in the respective clones were examined by Western blotting and immunofluorescent experiments and by patch clamping.

## Materials and methods

### Transfection of His-tagged prestin into CHO cells

The mammalian expression vectors developed in our laboratory was used [15]. Briefly, prestin cDNA cloned from gerbil cochlea, which was developed in our previous study [16], was used as a template. DNA fragments of His-tagged prestin were prepared by polymerase chain reaction (PCR). Such DNA fragments were inserted into two mammalian expression vectors, pBApo-EF1α Neo DNA and pBApo-CMV Neo DNA (Takara Bio, Shiga, Japan), using In-fusion cloning kit (Takara Bio).

The expression vectors were transfected into CHO cells using Lipofectamine 2000 (Thermo Fisher Scientific, Waltham, CA).

The Kanazawa University Safety Management Regulation for Genetic Recombinant Experiments, Kanazawa, Japan, approved the experimental protocols for this study (number:　Kindai6-2725). All procedures were completed in accordance with the relevant guidelines and regulations.

### Creation and cloning of a stable expression strain of prestin with His-tag

After transfection, CHO cells were cultured in RPMI-1640 medium (Sigma-Aldrich) with 10% fetal bovine serum (Biowest, Paris, France), 100 U penicillin/ml and 100 μg streptomycin/ml (Sigma-Aldrich) at 37°C with 5% CO_2_ for 2 days. Cells were then cultured for 3 weeks to 1 month in RPMI-1640 (Wako, Osaka, Japan) medium with 10% fetal bovine serum and geneticin (InvivoGen, SanDiego, CA; 600 μg/ml) at 37°C with 5% CO_2_ for drug selection. The culture media was changed every 2–3 days during this period.

After drug selection, cells were plated at a density of 1 cell/well in 96-well plates using the limiting dilution method. Three plates were used and incubated at 37°C with 5% CO_2_. Single colonies in 96-well plates were then scaled up. Cloned cells were collected for expression and quantification.

### Immunofluorescence experiments

To confirm the expression and localization of His-tagged prestin in the respective clones, immunofluorescence experiments were performed. CHO cells transfected with prestin and untransfected CHO cells were fixed with 4% paraformaldehyde in phosphate buffer at room temperature for 5 min and washed with PBS. Permeabilization was performed using Triton X-100 (Sigma-Aldrich). Cells were then incubated with 2% BlockAce (K.A.C., Kyoto, Japan) at room temperature for 30 min. After washing with PBS, permeabilized samples were incubated with an anti-His6 mouse monoclonal antibody (COSMOBIO, Tokyo, Japan) diluted 1:10000 in PBS at 4°C overnight. They were then washed with PBS and incubated with a Cy3 goat anti-mouse IgG secondary antibody (Themo Fisher Scientific) diluted 1:1000 in PBS at room temperature for one hour. Samples were then washed with PBS and immunofluorescence images were obtained using a fluorescent microscope (BZ-X810, KEYENCE, Osaka, Japan).

### Western blotting analysis

To confirm the full length of prestin, Western blotting analysis was performed. Briefly, SDS-PAGE was performed to CHO cells transfected with His-tagged prestin. CHO cells transfected with FLAG-tagged prestin [17] and multitope recombinant protein with a molecular weight of 74 kDa including 6×His (FUJIFILM Wako Pure Chemical Corporation, Osaka, Japan) were also loaded as negative and positive controls, respectively. After SDS-PAGE, it was blotted on a polyvinylidene difluoride (PVDF) membrane. The membrane was then treated with 5% skimmed milk (Wako, Tokyo, Japan) in PBS containing 0.05% Tween 20 (PBS-T) and incubated with anti-6×His-tag mouse monoclonal antibody (Proteintech, Rosemont, IL) diluted at 1:10,000 at 4°C overnight. Bands were visualized using a horseradish peroxidase (HRP)-linked anti-mouse IgG secondary antibody (Cell Signaling Technology, Danvers, MA) diluted at 1:5,000 and a chemiluminescent reagent (Amersham ECL Select Western Blotting Detection Reagent, Cytiva, Marlborough, MA).

### Electrophysiological experiments

To confirm the activity of prestin expressed in the respective clones, the electrophysiological properties of these clones were investigated. Prestin-expressing cells exhibit bell-shaped NLC in response to a change in membrane potential. Since NLC shows the voltage-dependent charge transfer of prestin, the anion transport function of prestin was evaluated by NLC. Patch pipettes for whole-cell patch-clamp recordings were pulled from borosilicate glass capillaries (World Precision Instruments, Sarasota, FL) with a puller (P-97, Sutter instrument, Novato, CA). Patch pipettes had a resistance of 2–5 MΩ when filled with an internal solution composed of 140 mM KCl, 3.5 mM MgCl_2_, 5 M EGTA, 5 mM HEPES, and 0.1 mM CaCl_2_, adjusted to pH 7.2. The external solution contained 145 mM NaCl, 5.8 mM KCl, 1.3 mM CaCl_2_, 0.9 mM MgCl_2_, 10 mM HEPES, 0.7 mM Na_2_HPO_4_ and 5.6 mM glucose, adjusted to pH 7.3.

**Fig 1:**
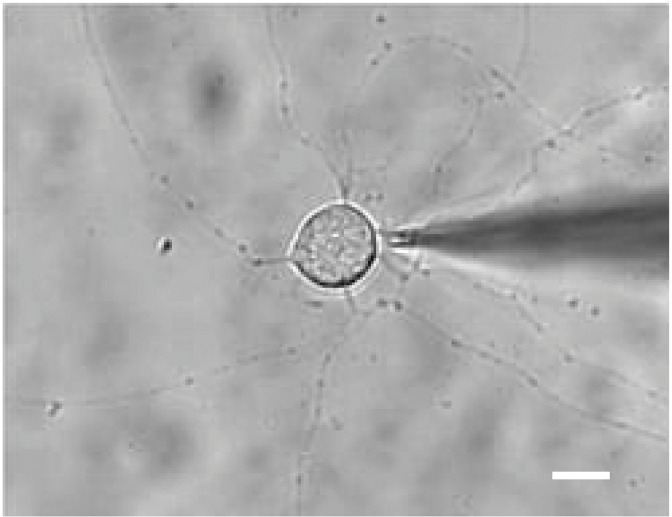
Microscopic image of a His-tagged prestin-transfected CHO cell from an electrophysiological experiment. The scale bar is 20 μm.

The measurement system consisted of a patch amplifier (Axopatch 200B amplifier, Axon instruments, Foster city, CA), an A/D and D/A converter (Digidata 1550A, Axon Instruments), a personal computer and a function generator (DF1906, NF Electronic Instruments, Kanagawa, Japan). Measurements of membrane capacitance were performed using the membrane test feature of Clampex 10.3 acquisition software (Axon Instruments). A test square wave (amplitude, 20 mV; period *T* = 40 msec, i.e., frequency, 25 Hz) was generated by the personal computer controlled by Clampex 10.3 software and applied to the cell through the amplifier (Fig 1). Transient current *Q*, current decay *τ* and total resistance *R*_*t*_ were continuously calculated by the software at a resolution of 25 Hz, by averaging the responses to 10 positive and 10 negative consecutive test steps, and measured values for these parameters were stored in the computer. Access resistance *R*_*a*_, membrane resistance *R*_*m*_ and membrane capacitance *C*_*m*_ were then obtained by substituting *Q*, *τ* and *R*_*t*_ into the following equations:

$$R_{a}=\frac{R_{t}\tau V_{c}}{QR_{t}+\tau V_{c}}$$ (1)

$$R_{m}=R_{t}-R_{a}$$ (2)

$$C_{m}={\left(\frac{R_{t}}{R_{m}}\right)}^{2}\frac{Q}{V_{c}}$$ (3)

where *V*_*c*_ is the voltage step. To assess the voltage dependence of membrane capacitance, triangular voltage ramps were superimposed on the above-mentioned square test wave. This triangular voltage wave (period *T* = 2 sec) was generated by the function generator and swung the cell potential from -140 mV to +70 mV. After measurements, membrane capacitance was plotted versus membrane potential.

Membrane capacitance recorded from prestin-expressing cells was fit to the derivative of the Boltzmann function,

$$C_{m}(V)=C_{lin}+\frac{Q_{max}}{{\alpha e^{\frac{V-V_{1/2}}{\alpha }}(1+e^{-\frac{V-V_{1/2}}{\alpha }})}^{2}}$$ (4)

where *C*_*lin*_ is linear capacitance, *Q*_*max*_ is maximum charge transfer, *V* is membrane potential and *V*_*1/2*_ is the voltage at half-maximal charge transfer. In Eq (4), α is the slope factor of voltage-dependent charge transfer and is given by

$$\alpha =\frac{kT}{ze}$$ (5)

where *k* is Boltzmann’s constant, *T* is absolute temperature, *z* is valence and *e* is the electron charge.

To estimate the NLC of the unit cell surface, normalized NLC *C*_*nonlin/lin*_ was defined as

$$C_{nonlin/lin}(V)=\frac{C_{nonlin}}{C_{lin}}=\frac{C_{m}(V)-C_{lin}}{C_{lin}}$$ (6)

The software program Kaleida Graph (Synergy Software, Reading, PA) was used for data analyses and curve fitting. To evaluate the expression level of prestin in the unit cell membrane, *Q*_*max*_, which reflects the expression level of prestin in the whole cell membrane, was normalized and designated as charge density. Normalization was achieved by dividing *Q*_*max*_ by *C*_*lin*_, which is proportional to the membrane area of cells. The unit of charge density is fC/pF.

### Statistical analysis

Immunofluorescence intensities were analyzed using one-way analysis of variance (ANOVA) followed by Dunnett’s *post hoc* multiple comparison. For electrophysiological experiments, statistical analyses were performed using one-way ANOVA. Data are presented in the text and Figures as the means ± SD. The p-value of less than 0.05 was considered significant. All statistical analyses were performed with EZR (Saitama Medical Center, Jichi Medical University, Saitama, Japan) [18], which is a graphical user interface for R (The R Foundation for Statistical Computing, Vienna, Austria).

## Results

### Stable expression of His-tagged prestin in CHO cells

We obtained 7 clones from the cells transfected with the expression vector including EF1α promotor and 5 clones from the cells transfected with the expression vector including CMV promotor. The expression of His-tagged prestin in CHO cells was examined by Western blotting and immunofluorescence experiments.

**Fig 2:**
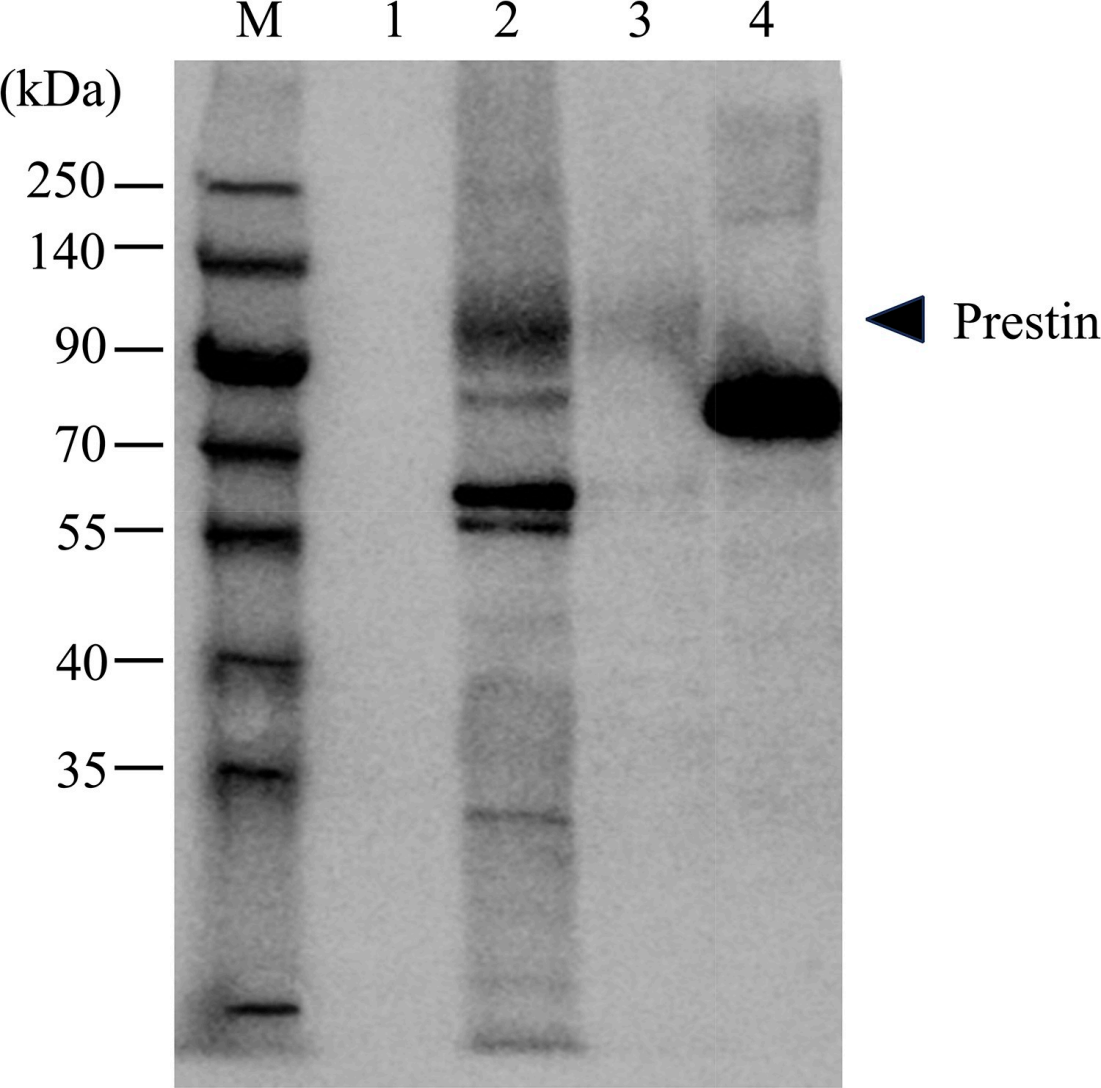
Western blot analysis of His-tagged prestin. Lane M: molecular weight marker. Lane 1: CHO cells transfected with FLAG-tagged prestin [17]. Lane 2: CHO cells transfected with His-tagged prestin (EF1α3C8). Lane 3: one-tenth the amount of sample in Lane 2. Lane 4: multitope recombinant protein including 6×His (internal and C-terminal) with a molecular weight of 74 kDa. A band at around 100 kDa in Lanes 2 and 3 represents His-tagged prestin.

**Fig 3:**
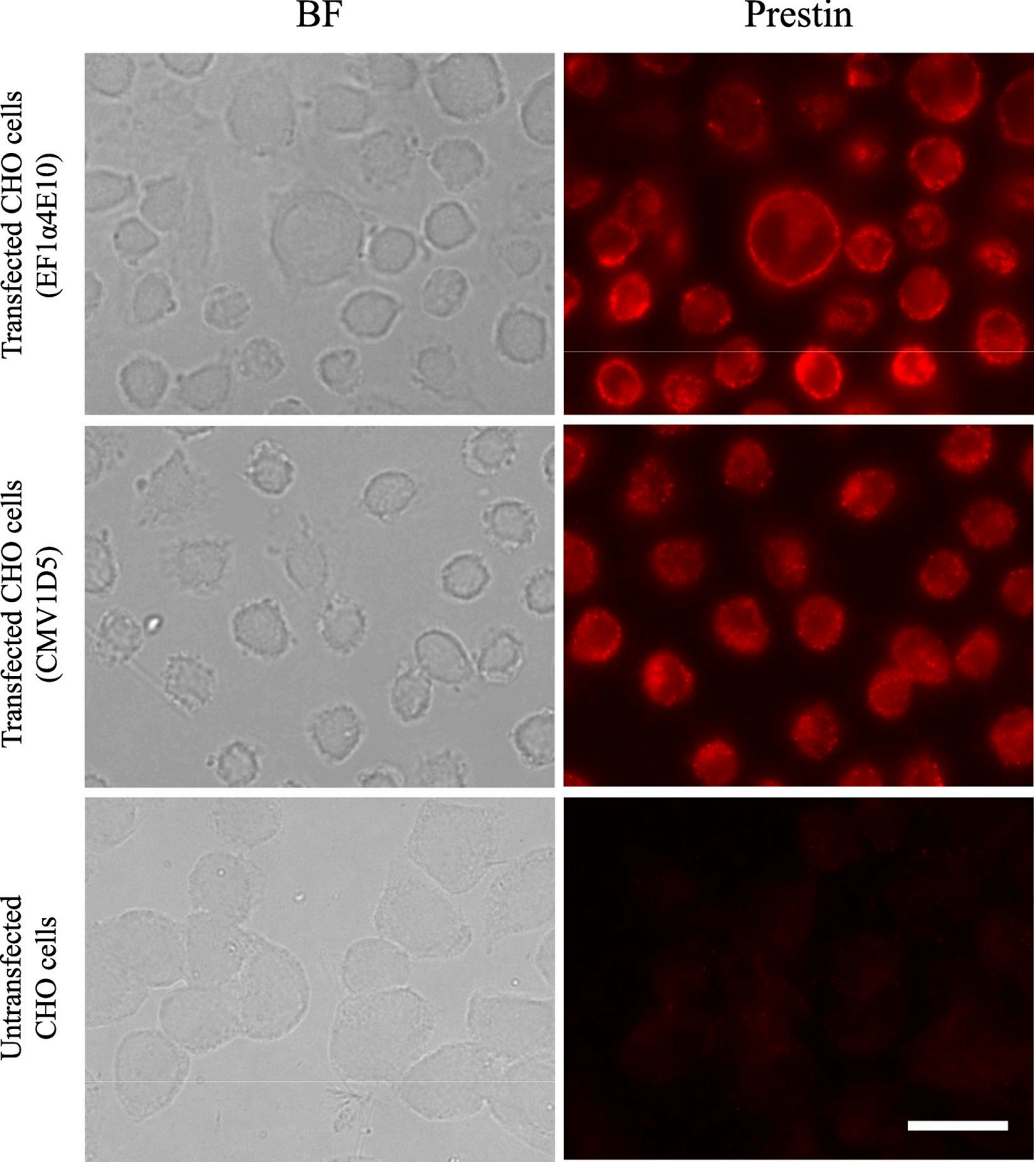
Bright field (BF) and fluorescence images of CHO cells. Upper panels, transfected CHO cells (EF1α4E10); middle panels, transfected CHO cells (CMV1D5); lower panels, untransfected CHO cells. Left panels, bright field (BF) images; right panels, prestin-labeled images. The expression of prestin was confirmed in transfected CHO cells. Scale bar is 20 μm.

**Fig 4:**
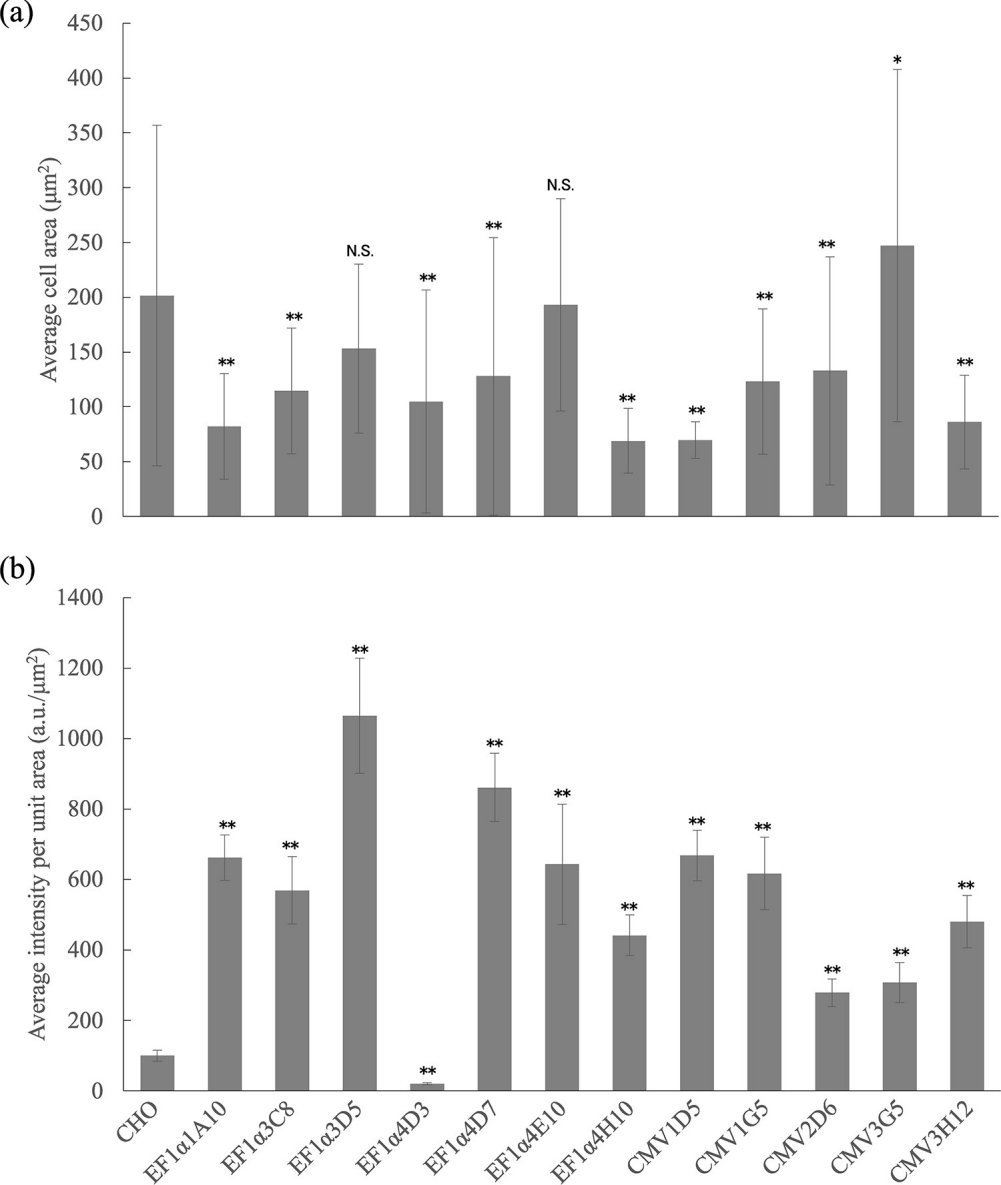
Cell area and fluorescence intensities of each constructed clone [20]. (a) Average cell area. Average cell area of CHO cells was not significantly different from those of EF1α3D5 and EF1α4E10 cells (N.S. *p* = 0.0865 and 0.9989 *re* CHO cells, Dunnett’s *post hoc* multiple comparison after one-way ANOVA). The other transfected cells, except for CMV3G5 cells, were significantly smaller than CHO cells (** *p* < 0.001 *re* CHO cells). In contrast, CMV3G5 cells were significantly larger than CHO cells (* *p* < 0.05 *re* CHO cells). (b) Average fluorescent intensities per unit area. Average intensities of the transfected CHO cells, except for EF1α4D3, were significantly larger than that of CHO cells (** *p* < 0.001 *re* CHO cells). In contrast, the intensity of EF1α4D3 cells was significantly smaller than that of CHO cells (** *p* < 0.001 *re* CHO cells).

Western blotting image is shown in Fig 2. Bands at around 100 kDa were observed from Lanes 2 and 3 (samples; CHO cells transfected with His-tagged prestin (EF1α3C8)) while it was not detected from Lane 1 (negative control; CHO cells transfected with FLAG-tagged prestin). In Lane 4 (positive control; recombinant protein including 6×His), a band at 74 kDa, corresponding to its molecular weight, was confirmed. Since the molecular weight of glycosylated prestin is about 100 kDa [17, 19], these results suggest that full-length prestin with His-tag was expressed in the developed cell line. Immunofluorescence images of a constructed cell line and untransfected CHO cells are shown in Fig 3. The average area and intensity in each clone are shown in Table 1. Average cell area and average fluorescence intensities per unit area of each clone are summarized in Fig 4. The average area of the transfected cells, except for EF1α3D5, EF1α4E10 and CMV3G5 cells, were smaller than that of CHO cells with statistical significance (** *p* < 0.001 *re* CHO cells). In contrast, CMV3G5 cells were larger than CHO cells (* *p* < 0.05 *re* CHO cells). Average intensities of the transfected CHO cells, except for EF1α4D3, were significantly larger than that of CHO cells (** *p* < 0.001 *re* CHO cells). In contrast, the intensity of EF1α4D3 cells was smaller than that of CHO cells (** *p* < 0.001 *re* CHO cells).

**Table 1:** Average area and intensity in constructed clones and untransfected CHO cells. The cumulative cell area and intensity of each clone were measured. Average intensity per unit area was obtained by dividing the latter by the former.

	*n*	Average cell area (μm^2^)	Average intensity per unit area (a.u./μm^2^)
CHO	65	201.45 ± 155.26	100.28 ± 15.48
EF1α1A10	121	82.23 ± 48.47	661.40 ± 64.26
EF1α3C8	123	114.44 ± 57.40	568.83 ± 95.74
EF1α3D5	29	153.14 ± 76.99	1064.56 ± 163.34
EF1α4D3	176	104.71 ± 101.72	20.45 ± 3.02
EF1α4D7	95	127.80 ± 126.63	860.98 ± 96.85
EF1α4E10	67	193.07 ± 96.86	642.78 ± 170.64
EF1α4H10	142	68.99 ± 29.43	441.15 ± 57.68
CMV1D5	115	69.59 ± 16.82	668.24 ± 71.99
CMV1G5	153	123.11 ± 66.19	616.56 ± 103.04
CMV2D6	159	132.94 ± 104.24	278.62 ± 39.46
CMV3G5	68	247.19 ± 160.63	307.37 ± 56.59
CMV3H12	145	86.08 ± 42.87	480.69 ± 74.35

### Functional analysis of cell line stably expressing prestin

**Fig 5:**
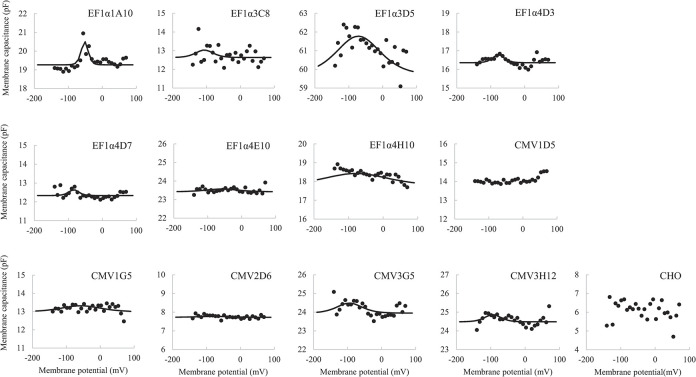
Membrane capacitance as a function of the membrane potential obtained from constructed clones. Data points are fit to the Boltzmann function (Eq (4)) and shown by solid lines [21].

**Fig 6:**
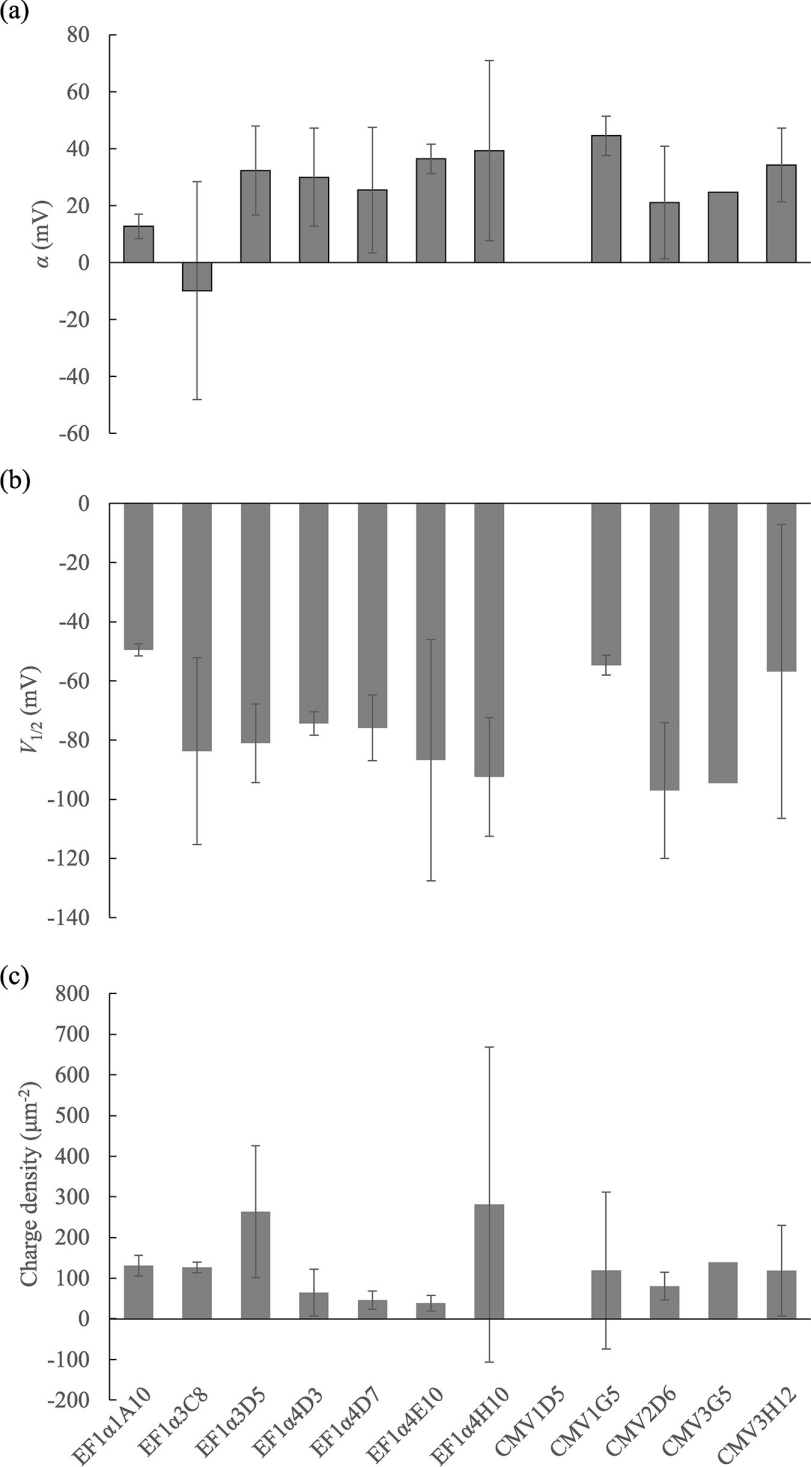
Fitting parameters of the derivative of the Boltzmann function [22]. (a) Slope factor α. (b) Voltage at half-maximal charge transfer *V*_1/2_. (c) Charge density α. These parameters were not significantly different between each clone (N.S. *p* = 0.386, 0.668 and 0.763 for α, *V*_1/2_ and charge density, respectively, one-way ANOVA).

The activity of prestin expressed in the generated cell line was examined by measuring NLC. Membrane capacitance versus membrane potential measured in the respective clones is shown in Fig 5. Clones with high fluorescent intensity (e.g. EF1α3D5) exhibited bell-shaped NLC. The fitting parameters of Eq (4) in the constructed clones are shown in Table 2 and Fig 6. The fitting parameters of Eq (4) in EF1α3D5, which showed the highest *Q*_*max*_ in the present study, were obtained as *C*_*lin*_ = 60.2±0.8 pF, *Q*_*max*_ = 253.2±153.2 fC, α = 43.4±15.6 mV, *V*_*1/2*_ = -71.7±13.2 mV, and charge density = 263.7±162.4 μm^-2^.

**Table 2:** Fitting parameters of the derivative of the Boltzmann function [22].

	C_lin_ (pF)	Q_max_ (fC)	α (mV)	V_1/2_ (mV)	Charge density (μm^-2^)
CHO (*n* = 2)	8.9±0.1	-	-	-	-
EF1α1A10 (*n* = 2)	19.2±0.1	40.0±7.6	12.8±4.3	-49.6±2.0	130.5±25.2
EF1α3C8 (*n* = 2)	12.0±1.0	24.4±4.4	-9.8±38.2	-83.7±31.6	126.7±13.0
EF1α3D5 (*n* = 2)	60.2±0.8	253.2±153.2	43.4±15.6	-71.7±13.2	263.7±162.4
EF1α4D3 (*n* = 3)	18.3±2.1	19.4±13.3	30.0±24.0	-74.4±0.6	64.4±57.7
EF1α4D7 (*n* = 2)	11.3±1.4	8.6±5.0	25.5±22.1	-75.8±11.0	45.9±22.3
EF1α4E10 (*n* = 2)	24.2±1.1	14.7±6.6	36.4±5.2	-86.8±40.7	38.4±18.8
EF1α4H10 (*n* = 2)	15.2±3.7	79.8±110.7	39.3±31.6	-92.5±20.0	281.6±387.6
CMV1D5 (*n* = 2)	17.8±5.3	N.A.	N.A.	N.A.	N.A.
CMV1G5 (*n* = 2)	14.2±1.7	24.4±40.5	44.5±6.9	-54.7±3.4	118.9±193.0
CMV2D6 (*n* = 2)	10.2±3.4	13.9±10.0	21.1±19.7	-97.1±22.9	80.1±34.3
CMV3G5 (*n* = 1)	23.9	53.3	24.8	-94.5	139
CMV3H12 (*n* = 3)	20.1±6.9	42.4±45.3	34.3±12.9	-56.8±49.7	118.1±111.6

## Discussion

In the present study, effects of two types of promoters, i.e., EF1α and CMV, on the expression of prestin in CHO cells were investigated. The CMV promoter is widely used for the expression of a target protein in a mammalian cell, being used in our previous works [16–18]. For the sake of the improvement of prestin expression, EF1α promoter was tested since it is often useful in conditions where the satisfactory expression could not be achieved by the other promoters.

The expression level of prestin in the respective clones was estimated using linear capacitance *C*_*lin*_ and maximum charge transfer *Q*_*max*_. Since *Q*_*max*_ indicates the total amount of charge carried by all prestin in the cell membrane and *e* is the electron charge, which is presumed to equal the charge carried by one prestin molecule, the number of prestin molecules in the cell is given by *Q*_*max*_/*e*. *C*_*lin*_ indicates the total capacitance of the cell membrane, and the membrane capacitance of a cell per unit surface area is known to be 0.01pF/μm^2^ [23]; therefore, the surface area of a cell is expressed by *C*_*lin*_/0.01 μm^2^. The expression level of prestin per unit surface area, i.e., charge density, of a cell may be obtained as follows:

$$Charge\;density=\frac{Q_{max}}{e}/\frac{C_{lin}}{0.01}$$ (7)

The fluorescent intensities of the constructed clones measured by immunofluorescence experiments showed similar results to those on the charge densities of these clones measured by patch clamping. The expression of prestin was confirmed by the fluorescence emitted from CHO cells. Cells exhibiting this fluorescence showed higher charge densities. However, NLC was not detected in some clones (e.g., EF1α4E10 and CMV 1D5) even though high fluorescent intensities were observed.

The *α* is a slope factor that characterizes voltage-dependent membrane capacitance and also shows the properties of anion binding and transport. A larger *α* value shows that a larger potential change is necessary for anion binding and translocating anions across a cell membrane. *V*_*1/2*_ is the voltage at which charges are moved with the smallest voltage increment. The shift of *V*_*1/2*_ indicates that the point at which prestin is sensitive to the membrane potential. In the constructed clones, *α* and *V*_*1/2*_ were considered to have little effect on the function of prestin.

**Fig 7:**
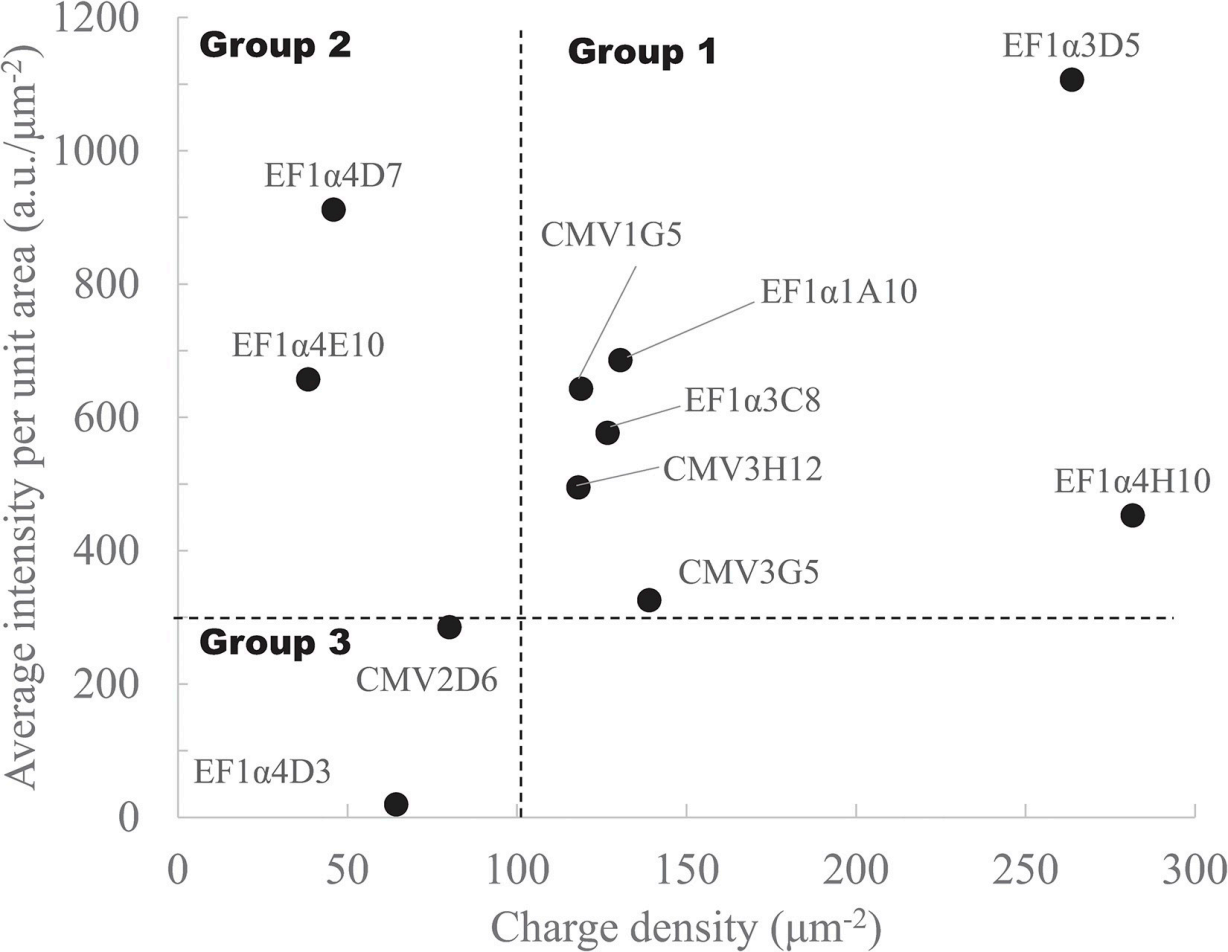
Relationship between the average intensity per unit area and charge density. Clones were divided into 3 groups based on immunofluorescent intensities and charge densities.

We divided 11 clones (except for CMV1D5, for which charge density was not available) into 3 groups based on their immunofluorescent intensities and charge density (Fig 7): a high intensity and high charge density group (group 1: EF1α1A10, EF1α3C8, EF1α3D5, EF1α4H10, CMV1G5, CMV3G5, and CMV3H12), a high intensity and low charge density group (group 2: EF1α4D7 and EF1α4E10), and a low intensity and low charge density group (group 3: EF1α4D3 and CMV2D6). In group 1, prestin was expressed in the cell membrane and functioned well. In group 2, prestin was expressed in CHO cells and was not functional or some prestin was not expressed in cell membranes and remained in the cytoplasm. In group 3, prestin was not considered to be expressed in the CHO cell membrane. The cells transfected with EF1α promoter tended to show higher expression level compared with those transfected with CMV promoter. On the other hand, as for the charge density, it varied from the lowest level (group 2) to the highest level (group 1) in the cells transfected with EF1α promoter, suggesting that the expressed prestin was somehow functionally unstable in this expression system. When selecting appropriate clones from among them, therefore, both immunological and physiological evaluations are indispensable.

In the present study, we attempted to evaluate the functionality of prestin using NLC, which is widely recognized as a measure of prestin function [6, 7, 24]. Although there is a possibility that prestin has received some different post-translational modification from OHCs, the results of this study indicate that prestin is functional in this expression system and may be received similar post-translational modification. Regarding glycosylation, which is one type of post-translational modification, for example, it still functions in our expression system because it is known that its improper modification leads to disappearance of NLC. Furthermore, western blotting analysis of this cell line showed a prestin band near 100 kDa (data not shown). It is slightly larger than the molecular weight of prestin (81.4 kDa) and suggests the presence of glycosylated prestin [19].

The charge densities of the constructed clones were lower than the average charge densities of OHCs; i.e., approximately 118.1 (CMV3H12) - 281.6 (EF1α4H10) μm^-2^ in the constructed CHO cell line and 7500 μm^-2^ in OHCs reported in a previous study [5]. Although charge density was approximately 1/60–1/30 of those in OHCs, cell line maintaining the stable expression of full-length prestin will be advantageous for future analyses of its structure and function.

Overall, in the present study, stable expression systems for prestin with His-tag were constructed and prestin expressed in these systems was confirmed to be functional by patch clamping. Although function levels in the constructed cell line were 1/60–1/30 of those in OHCs, His-tagged prestin will contribute to future investigations on the structure and function of prestin.
